# Formulation and Characterisation of an Edible Functional Elderflower Extract Gel for Upper Respiratory Tract Soothing

**DOI:** 10.3390/gels12040272

**Published:** 2026-03-26

**Authors:** Jurga Andreja Kazlauskaite, Daiva Majiene, Giedre Kasparaviciene, Lina Raudone, Inga Matulyte

**Affiliations:** 1 Department of Drug Technology and Social Pharmacy, Lithuanian University of Health Sciences, LT-50161 Kaunas, Lithuania; jurga.andreja.kazlauskaite@lsmu.lt (J.A.K.); daiva.majiene@lsmu.lt (D.M.); giedre.kasparaviciene@lsmu.lt (G.K.); 2 Institute of Pharmaceutical Technologies, Lithuanian University of Health Sciences, LT-50161 Kaunas, Lithuania; lina.raudone@lsmu.lt; 3 Laboratory of Biochemistry, Neuroscience Institute, Lithuanian University of Health Sciences, LT-50162 Kaunas, Lithuania; 4 Department of Pharmacognosy, Lithuanian University of Health Sciences, LT-50162 Kaunas, Lithuania

**Keywords:** elderflower, polyphenols, gel, respiratory tract, soothing

## Abstract

The increasing demand for natural functional products for soothing the upper respiratory tract has stimulated interest in plant-based bioactive formulations with antioxidant and cytoprotective properties. This study aimed to develop and characterise an edible gel containing elderflower extract and to evaluate its physicochemical properties and biological activity in an in vitro inflammatory model. The extract was characterised in terms of total phenolic content (TPC), antioxidant capacity (FRAP, ABTS, DPPH), and compound composition using chromatographic analysis. The gel formulation was assessed for texture parameters and pH. Biological activity was evaluated using HBEC-3 cells exposed to lipopolysaccharide (LPS, 1 µg/mL) for 24 h, followed by treatment with the extract in free or gel-incorporated form at different concentrations. The elderflower extract exhibited high TPC and strong antioxidant activity, with chlorogenic acid, rutin, and quercetin derivatives identified as major phenolic compounds. The formulated gel showed suitable firmness, consistency, cohesiveness, and a mildly acidic pH. LPS exposure reduced cell viability by approximately 48%, whereas treatment with the extract significantly increased viability in a concentration-dependent manner. At the highest tested concentration (50 mg/mL), the gel increased cell viability by 36% compared to LPS alone and markedly reduced necrotic cell death. These results indicate that the formulated edible elderflower extract gel combines favourable physicochemical properties with concentration-dependent cytoprotective effects, supporting its potential application for upper respiratory tract soothing.

## 1. Introduction

Elderflower (*Sambucus nigra* L.) is also known as elder, black elder, elderberry, or European elder, a species from the *Viburnaceae* family [1,2]. Elderflower remains widely used in traditional practices across various countries for the treatment and prevention of colds, influenza, and other upper respiratory tract infections [3,4]. Elderflower triterpenes stimulate mucus secretion and the cough reflex, while other constituents stabilise blood vessels and strengthen mucosal barriers, thereby reducing the risk of infection. These characteristics make elderflower extract a promising functional ingredient for formulations intended to soothe irritated respiratory tissues [4,5].

Elderflowers contain a variety of bioactive compounds, such as flavonoids, phenolic acids and their glycosides—including caffeic, ferulic, chlorogenic, and p-coumaric acids—as well as triterpenes like α- and β-amyrin and ursolic acid, sterols, and essential oils [6]. Several of these compounds exhibit antioxidant, anti-inflammatory, and mucosal-protective properties [5,6]. Notably, elderflower triterpenes are known to promote mucus secretion and activate the cough reflex. Additionally, other components might help stabilise blood vessels and reinforce mucosal barriers, which could lower the risk of infection [6]. Due to their potent antioxidant properties, compounds from elderflowers may enhance cellular defences against oxidative stress, which plays a role in inflammation and respiratory irritation [4,6,7].

The efficacy of plant-derived active ingredients primarily depends on the delivery method. Oral delivery is vital for soothing the respiratory tract because it allows direct contact with the oral and pharyngeal mucosa [8]. This contact can help form a protective layer on irritated tissues, aiding in hydration, strengthening the barrier, and enhancing local comfort. Edible formulations that stay in the mouth long enough can offer soothing benefits in the upper respiratory tract while also enabling systemic absorption of bioactive compounds after swallowing [9]. Gels form a semi-solid matrix that can protect delicate phytochemicals, enable the controlled release of active ingredients, and enhance consumer appeal. Additionally, using food-grade gelling agents ensures that these formulations are safe for oral intake while still delivering functional benefits [9,10,11]. These features make gels an excellent choice for incorporating elderflower extracts into edible products.

Although much research has examined the phytochemical composition and biological effects of elderflower extracts, limited studies have explored their use in edible gel systems. In particular, little is known about the formulation, physicochemical properties, and functional applications of elderflower extract hydrogels intended for oral consumption and respiratory relief. This study, therefore, aimed to develop and characterise a functional elderflower extract hydrogel for oral use, aimed at soothing the upper respiratory tract. The objective is to support the development of natural, well-structured gel products for respiratory comfort, with a focus on formulation details, physicochemical properties, and biological safety.

## 2. Results and Discussion

### 2.1. Antioxidant Properties and Phytochemical Profile of the Extract

The elderflower extract showed a high total phenolic content (TPC) of 197.75 ± 10.54 mg GAE/g, indicating it is a rich source of polyphenolic compounds. Its antioxidant capacity was consistently strong across multiple assays (Table 1). These results show that the extract possesses both strong reducing power and efficient free radical neutralising ability. Compared with other studies on Sambucus nigra flower extracts, the TPC of our extract (197.7 mg GAE/g) exceeds previously reported ranges (82.4–157.3 mg GAE/g) for ethanol and water extracts influenced by extraction method and geographical origin [12].

The high TPC measured in our extract is consistent with previous studies that identified elderflowers as one of the richest plant sources of phenolic compounds. Mikulic-Petkovsek et al. reported that elderflowers contain significantly higher levels of phenolics compared to elderberries, which is reflected in their stronger antioxidant activity [7].

**Figure 1:**
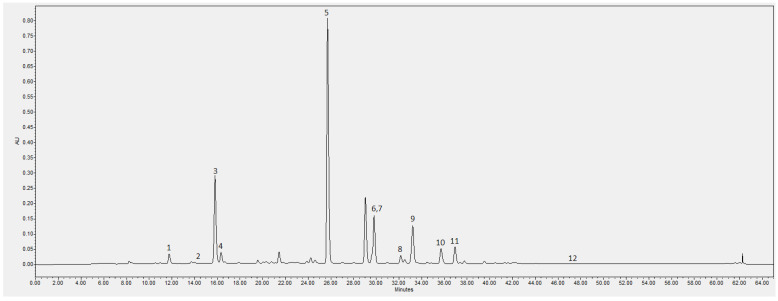
The elderflower extract compounds chromatogram. 1—Neochlorogenic acid; 2—protocatechuic acid; 3—chlorogenic acid; 4—4-o-caffeoylquinic acid; 5—rutin; 6—isoquercitrin; 7—nicotiflorin; 8—4,5-di-caffeoylquinic acid; 9—astragalin; 10—3,5-di-caffeoylquinic acid; 11—3,4-di-caffeoylquinic acid; 12—quercetin.

Phytochemical analysis revealed several phenolic compounds contributing to the antioxidant activity. Among these, rutin and chlorogenic acid were the most abundant, followed by isoquercitrin, quercetin, kaempferol, and cyanidin-3-glucoside (Figure 1, Table 2).

The phenolic composition of the extract was dominated by rutin (3127.24 µg/mL) and chlorogenic acid (3768.16 µg/mL), consistent with earlier research describing these compounds as the primary phenolics in elderflower [6,13].

Rutin, quercetin, and caffeoylquinic acids are particularly well known for their radical-scavenging properties due to their catechol structure, which facilitates electron donation. They also possess anti-inflammatory effects mediated through suppression of nitric oxide production and modulation of redox-sensitive signalling pathways [7,14,15,16]. Chlorogenic acid, another abundant phenolic, is also a recognised contributor to both antioxidant and anti-inflammatory activities [17]. The high concentration of these compounds in the extract likely explains the pronounced FRAP activity, as this assay primarily reflects reducing capacity under acidic conditions. The substantial levels of flavonol glycosides such as rutin and isoquercitrin further support the antioxidant effects, as these compounds are capable of scavenging reactive oxygen species and chelating transition metals [18,19].

Differences among FRAP, ABTS, and DPPH assays arise from their unique reaction mechanisms. FRAP assesses the reducing power of ferric ions (Fe^3+^) in acidic conditions, while ABTS and DPPH detect radical scavenging via electron and hydrogen transfer [20]. Assay-dependent variability is frequently reported for complex botanical extracts due to structural diversity, redox potential differences, and steric effects of individual phenolics, which may explain the comparatively lower DPPH value observed in the present study [21].

### 2.2. Results of Gel Preparation and Texture Analysis

**Figure 2:**
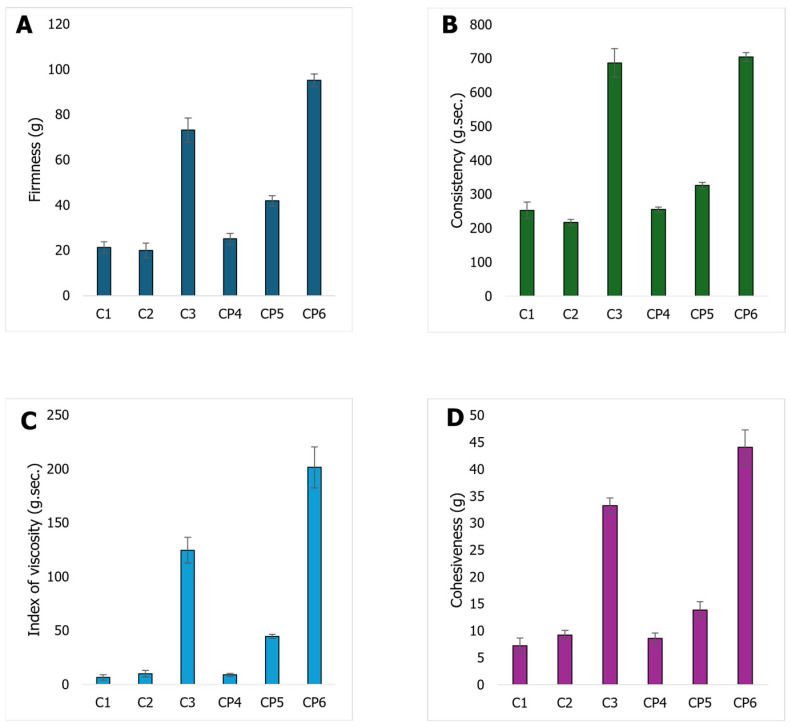
Texture parameters of gels (**A**) firmness; (**B**) consistency; (**C**) index of viscosity; (**D**) cohesiveness.

The xanthan gum–glucomannan ratio was selected based on preliminary optimisation experiments, where this combination provided the most favourable balance between firmness, structural integrity, and suitability for oral application. After incorporation of the elderflower extract, the formulation was intentionally not re-optimised in order to isolate the direct impact of the extract on gel network behaviour. Therefore, the observed decrease in hardness and cohesiveness reflects intrinsic polymer–extract interactions rather than secondary compositional adjustments. Based on the evaluation of the gels’ mechanical property parameters (Figure 2) and other properties, the CP6 gel was selected. In this formulation, ethanol was replaced with elderflower extract.

The addition of a preservative to the gel formulations demonstrated that potassium sorbate influenced the mechanical properties and pH value of the gels—formulations containing the preservative were firmer, more viscous, and exhibited improved consistency compared to gels without preservatives. Evaluation of the pH values revealed that the gel C1 had the lowest pH (pH = 3.8), while CP6 exhibited the highest pH (pH = 4.15). Potassium sorbate is an effective preservative that not only inhibits the growth of microorganisms, but can also modify the physical and chemical properties of products through pH and network interactions [10]. According to Kowalczyk et al. (2020), potassium sorbate incorporation into edible films influenced their physicochemical and mechanical properties, suggesting that the preservative may affect polymer network interactions and structural organisation, particularly under pH-dependent conditions [22]. The final gel has a mildly acidic pH (4.46 ± 0.15). This range is generally compatible with oral consumption from a tolerability standpoint and is aligned with many edible/nutraceutical gel-type products. From a mucosal tolerability perspective, pH 4–5 is commonly encountered in foods and is not inherently unusual for oral products. In pharmaceutics, oral liquid products are often formulated across a broad pH window as a compromise among stability, preservative efficacy, and acceptability; literature reviews note that pH values roughly between ~2 and 9 can be tolerated depending on composition and exposure conditions [23].

In this study, the gel is not “strongly acidic”, which improves expected tolerability compared with highly acidified products [24]. Published work on gummy-type products reports typical pH values in the ~3–5 range, with specific examples showing candies around pH 3.54–3.91 and explicitly stating that these values align with the typical pH of gummies (3–5) [25]. Therefore, pH 4.46 sits comfortably within the reported “gummy/edible gel” domain, and is close to common target ranges used to balance taste, microbiological robustness, and ingredient stability.

Although a pH of approximately 4.5 is typical for edible gel and nutraceutical products, it is below the critical enamel demineralisation threshold (~5.5). Therefore, similar to other mildly acidic foods, frequent or prolonged oral exposure may increase the risk of dental erosion, particularly if the product is retained in the mouth for extended periods or consumed repeatedly throughout the day [26]. To minimise this risk, it is recommended to avoid prolonged oral retention and to consume the gel with water or during meals.

The combination of xanthan gum and glucomannan used in gel formulation proved to be the most suitable, resulting in a gel with the most favourable properties, particularly appropriate for potential oral use. Xanthan gum and konjac glucomannan mixtures demonstrate a synergistic gelation effect, resulting in significant improvements in the mechanical and rheological properties of the gel—such as strength, stiffness and consistency [27]. According to Günter et al. (2024), the combination of xanthan gum and konjac glucomannan in hydrogel systems resulted in improved mechanical properties, such as hardness, cohesiveness, and gumminess, with the effect becoming more pronounced at increased polysaccharide concentrations, indicating synergistic interactions between the polymers [28].

The influence of elderflower extract on the properties of the gel was evaluated, and the results are presented in Table 3.

**Figure 3:**
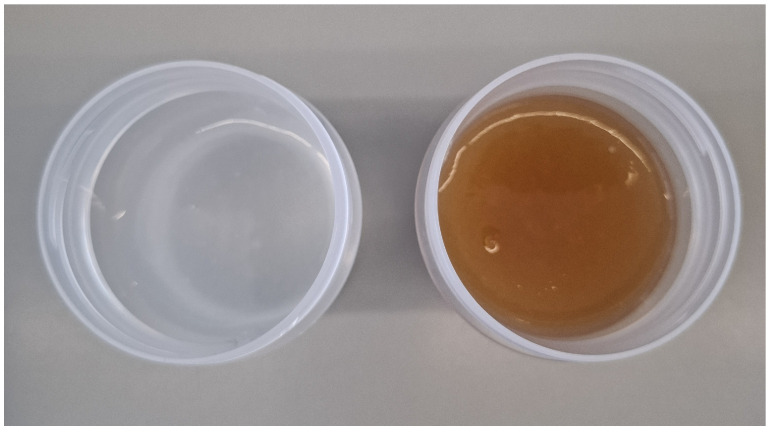
CP6—colorless transparent gel base (**left**); G—brown gel with plant extract (**right**).

The texture properties of the gel containing elderflower extract, compared to the control gel (CP6) (Figure 3), showed the following differences: firmness was 17.68% lower, consistency was 5.5% higher, cohesiveness decreased 3.14 times, and the index of viscosity was 3.7 times lower. The pH value of the gel containing elderflower extract was 7.2% higher compared to the control gel. Plant extracts, rich in phenolics, polysaccharides, and proteins, contribute to the gelation process through various mechanisms, enhancing the three-dimensional gel network by hydrogen bonding and other interactions, which influence elasticity, viscosity, and stability [10].

The structural modifications observed following extract incorporation can be explained by established polyphenol–polysaccharide interaction mechanisms. Phenolic compounds are known to form non-covalent interactions, particularly hydrogen bonding and hydrophobic associations, with hydroxyl-rich polysaccharides. Such interactions may competitively interfere with polymer–polymer associations, reducing effective crosslink density within hydrocolloid networks and consequently weakening gel strength and cohesiveness [29,30].

In addition, low-molecular-weight extract constituents may exert a plasticising effect by increasing polymer chain mobility, while soluble components can modify water distribution and hydration dynamics within the matrix, further influencing gel structure and mechanical parameters [31]. The pH range of the studied gels (3.8–4.15) is also relevant for structural regulation. Xanthan gum contains glucuronic acid residues with reported pKa values around 3.1–3.5; thus, partial ionisation of carboxyl groups occurs under these mildly acidic conditions, influencing electrostatic repulsion and intermolecular association within the network [31]. Even minor pH variations may alter xanthan chain conformation and affect the architecture of the xanthan–glucomannan synergistic system.

Furthermore, potassium sorbate (pKa ≈ 4.8) exists in a partially dissociated state at this pH. Variations in ionic strength are known to modulate intermolecular spacing and rheological behaviour in charged polysaccharide systems through electrostatic screening effects [30,32]. Collectively, polymer–polyphenol interactions, pH-dependent ionisation phenomena, and ionic effects induced by the preservative provide a coherent physicochemical explanation for the observed reductions in hardness, cohesiveness, and viscosity index after extract incorporation.

Furthermore, the concentration of bioactive extracts influences hydrogel density and swelling behaviour, which in turn affects mechanical parameters such as dimensional stability and viscoelasticity [33]. The stability was assessed over roughly two months. During this time, there were no notable changes in pH, texture parameters, or visual appearance.

### 2.3. Effect of LPS, Gel Formulation, and Elderflower Extract on the Viability of HBEC-3

Previous reports indicate that elderflower extracts do not adversely affect cell viability at low concentrations, remaining within a non-cytotoxic range, which is consistent with observations showing unchanged viability at minimal doses [34].

**Figure 4:**
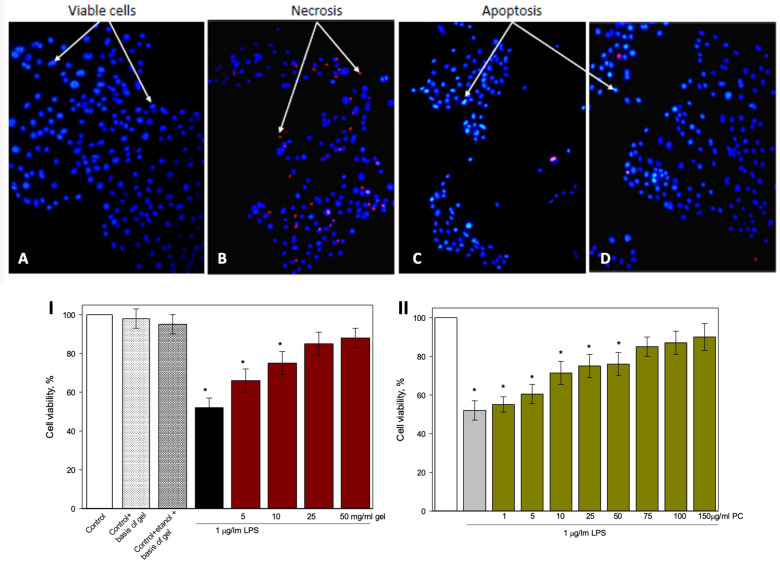
Effect of formulations on the viability of human bronchial epithelial cells (HBEC-3). Representative micrographs (magnification ×320): (**A**) untreated control; (**B**) cells treated with LPS (1 µg/mL, 24 h); (**C**) LPS (1 µg/mL) + gel (10 mg/mL); (**D**) LPS (1 µg/mL) + gel (50 mg/mL). Quantitative analysis: (**I**) effect of gel on cell viability; (**II**) effect of extract on cell viability. Data are presented as mean ± SE. * *p* < 0.05 versus control.

The extract concentration used in this study was determined based on previous results [35]. Exposure of HBEC-3 cells to 1 µg/mL lipopolysaccharide (LPS) for 24 h reduced cell viability by approximately 48% (Figure 4I,II). In the affected wells, a markedly lower cell density was observed, with a large proportion of cells undergoing necrotic death (Figure 4B). HBEC-3 cells constitute a well-established human airway epithelial model that accurately replicates essential processes such as maintaining epithelial barrier integrity and mediating inflammatory signaling involved in mucosal irritation and soothing. Although derived from bronchial tissue, both upper and lower airway epithelia share common defense mechanisms, including cytokine release, responses to oxidative stress, and epithelial repair, which are pertinent to the biological effects under investigation [36,37,38].

Treatment with the gel formulation containing elderflower extract at the lowest tested concentration had no significant effect on cell viability (Figure 4I). However, higher concentrations of the gel significantly increased viability: more viable cells were observed, necrotic death was markedly reduced, and a relative increase in apoptotic cells was detected, indicating a shift toward a more favourable cell death pathway (Figure 4C). At the highest tested concentration (50 mg/mL), the gel increased viability by 36% compared to LPS alone (Figure 4D), with images showing a substantially higher number of viable cells and only a few apoptotic ones. Similar cell culture studies have shown that elderflower (*Sambucus nigra*) extracts can stimulate cell proliferation and viability, even under inflammatory challenge conditions induced by LPS, suggesting a protective effect that mitigates cytotoxic stress and supports cellular health [39]. Based on Ferreira at al. (2022) study, elderflower extracts have been reported to reduce intracellular oxidative stress and protect against oxidative damage in cell models, potentially supporting cell survival and reducing necrotic pathways [34].

Similar results were obtained with the elderflower extract tested across a range of concentrations (1–150 µg/mL PC) on LPS-treated cells (Figure 4II). The lowest concentrations (1 and 5 µg/mL PC) did not affect viability. Intermediate concentrations (10–50 µg/mL PC) increased viability by 16–21%, while higher concentrations (75–150 µg/mL PC) enhanced viability by 33–38%.

Importantly, the effect of elderflower extract on cell viability was dependent on concentration but independent of formulation. In other words, when applied at the same concentration of active compounds, the extract in free form or incorporated into the gel exerted an equivalent effect on HBEC-3 viability. Several studies have reported that the biological activity of phenolic-rich plant extracts is primarily determined by the concentration of active compounds rather than the formulation matrix, with comparable cellular responses observed for free extracts and extracts incorporated into hydrogel or polymer-based delivery systems [40,41].

Compared to traditional nutraceutical forms like syrups, lozenges, and capsules, a hydrogel system is more effective for targeted mucosal delivery because it creates a moist matrix at the application site. When mucoadhesive, it helps prolong retention on mucosal tissues, which is especially important in active environments like the mouth and upper aerodigestive tract, where quick clearance occurs due to saliva and mechanical actions forces [42,43].

Many hydrogel drug-delivery systems are designed for controlled or sustained release, commonly used for topical wound treatment, injectable depots, or localized delivery in various tissues. However, delivering drugs to the oral or oropharyngeal mucosa presents unique challenges, such as a wet surface, salivary washout, and frequent movement. These constraints have led to a focus on adhesive and mucoadhesive hydrogels, as well as other localized delivery systems tailored specifically for oral, maxillofacial, or oral mucosal applications [42,44,45].

Nevertheless, while hydrogels can improve local retention and potentially support local delivery, the present work remains in vitro and therefore cannot fully capture the complexity of mucosal clearance, irritation triggers, or host responses in vivo. We therefore position these findings as an initial formulation and screening step, and acknowledge that further validation is required to benchmark performance against advanced oral mucosal delivery platforms described in the literature [44,46].

## 3. Conclusions

This study successfully formulated and characterised an edible gel containing elderflower extract, focusing on its antioxidant activity, texture, and biological effects. The extract demonstrated high total phenolic content and potent antioxidant capacity, with phenolic acids and flavonoids, such as chlorogenic acid, rutin, and quercetin derivatives, contributing to these properties. The gel showed appropriate firmness, consistency, cohesiveness, viscosity, and a mildly acidic pH, indicating suitable physicochemical qualities for potential oral application.

In vitro tests revealed that exposure to LPS significantly decreased HBEC-3 cell viability and caused necrotic death. Treatment with elderflower extract enhanced cell viability in a concentration-dependent manner. Lower extract doses had no effect, whereas higher doses notably improved cell survival and reduced necrosis. These effects were consistent whether the extract was applied freely or embedded in the gel, demonstrating that the formulation did not diminish its biological activity.

Overall, the data indicate that this elderflower extract gel possesses beneficial physicochemical characteristics and offers protective effects that elevate concentration in inflammatory conditions, emphasising its potential as a soothing treatment for the upper respiratory tract. Future research should incorporate in vivo testing to verify safety and effectiveness in conditions that mimic the physiological environment, followed by clinical validation to evaluate real-world performance. Moreover, additional formulation refinement and stability testing will be required to facilitate regulatory approval and support the possible commercialization of the hydrogel system.

## 4. Materials and Methods

### 4.1. Plant Material and Reagents

The raw plant material of *Sambucus nigra* L. (dry flowers) was obtained from “Emili” (Vilnius, Lithuania) in 2023. In this experiment, water was purified using a Milli–Q system (Millipore, Bedford, MA, USA). Ethanol (96%) was obtained from “Vilniaus degtine” (Vilnius, Lithuania). Anhydrous acetic acid (99.8%), hydrochloric acid (37%), and HPLC standards were purchased from Sigma-Aldrich (Buchs, Switzerland). Cell culture reagents were obtained from Gibco (Fisher Scientific, Paisley, UK). All the reagents and standards were of analytical grade. Folin–Ciocalteu phenol reagent (Merck, Darmstadt, Germany). 2,2′-azino-bis(3-ethylbenzothiazoline-6-sulfonic acid) (ABTS), TPTZ (2,4,6-tri-2-pyridinyl-1,3,5-triazine), 2,2-diphenyl-1-picrylhydrazyl radical (DPPH) purchased from Sigma Aldrich (Hamburg, Germany).

### 4.2. Preparation of a Hydroethanolic Elderflower Extract via Percolation

To produce 40 g of elderflower extract, 40 g of dried flowers were extracted with 400 mL of 50% (*v*/*v*) ethanol. The process began by mixing the plant material with 100 mL of 50% ethanol and subjecting it to ultrasonic treatment for 15 min. The mixture was then transferred to a percolator and extracted over three days.

At the end of the percolation process, the extract was divided into two parts. The first part contributed 80% of the total yield, which was 32 g (part 1). The second part was obtained by extracting the swollen raw material with the remaining 300 mL of solvent until total washout, resulting in a volume 5 to 8 times larger than part 1. This second part of the extract was concentrated by heating on the heating plate at the solvent’s boiling point (85 °C) and by evaporation until a thick mass formed. It was then combined with part 1 to produce the final 40 g of liquid extract, which was used in subsequent studies.

### 4.3. Analysis of the Chemical Composition

#### 4.3.1. Total Phenolic Compound (TPC) Analysis

The total phenolic content was determined using the Folin–Ciocalteu colorimetric assay. A 10 µL of the sample extract was mixed with 1.590 µL of distilled water and 100 µL of Folin–Ciocalteu reagent. After an incubation period of 6 min, 300 µL of 20% sodium carbonate solution was added to the mixture. The reaction mixture was subsequently incubated at room temperature for 2 h. Absorbance was measured at 760 nm using a Thermo Scientific Fluoroskan Ascent spectrophotometer (Thermo Scientific, Vantaa, Finland). The results were calculated and reported as milligrams of gallic acid equivalents per gram of extract (mg GAE/g) [47].

#### 4.3.2. High-Performance Liquid Chromatography with Photodiode Array Detection (HPLC–PDA) Analysis

High-performance liquid chromatography (HPLC) analysis was carried out using a Waters e2695 Alliance HPLC System (Waters, Milford, MA, USA) equipped with a Waters 2998 Photodiode Array Detector for the determination of phenolic compounds. Separation was achieved on an ACE C18 HPLC Column (150 mm × 4.6 mm, particle size 3 μm; Advanced Chromatography Technologies (ACT), Aberdeen, UK).

The mobile phase consisted of solvent A (0.05% trifluoroacetic acid) and solvent B (acetonitrile) applied in a gradient program: 0–5 min—12% B, 5–50 min—12–30% B, 50–51 min—30–90% B, 51–56 min—90% B, and returning to 12% B at 57 min. The flow rate was maintained at 0.5 mL/min, and the injection volume was set at 10 μL.

Compound identification was based on comparison of retention times and UV absorption spectra between analytes and corresponding reference standards. Calibration curves were prepared using standard solutions. Quantification of phenolic acids was performed at 325 nm, whereas flavonoids were determined at a wavelength of 350 nm.

### 4.4. Antioxidant Activity of the Elderflower Extract

#### 4.4.1. Ferric Reducing Antioxidant Power (FRAP) Assay

The working FRAP reagent was prepared by mixing 10 volumes of 300 mM acetate buffer (pH 3.6), 1 volume of 10 mM TPTZ (2,4,6-tri(2-pyridyl)-s-triazine) in 40 mM HCl, and 1 volume of 20 mM FeCl_3_. All solutions were freshly prepared prior to use. For the assay, 100 µL of sample (mg/mL) was combined with 3 mL of FRAP reagent and incubated for 6 min at room temperature. Absorbance was measured at 593 nm against a blank. FRAP values were calculated from the difference in absorbance between the sample and blank, and results were expressed as mg Fe(II)/g of sample, based on a ferric chloride calibration curve (0–100 mg/g; y = 2.6272x, R^2^ = 0.9985).

#### 4.4.2. ABTS Radical Scavenging Activity Assay

The ABTS radical cation was produced by combining 7 mM ABTS with 2.45 mM potassium persulfate at a molar ratio of 1:0.5, followed by incubation in the dark for 12–16 h. The resulting solution was subsequently diluted with water until an absorbance of 0.90 ± 0.02 at 734 nm was obtained.

For the determination, 2 mL of the prepared ABTS solution was mixed with 200 µL of the sample and kept in the dark for 30 min. After incubation, absorbance was recorded at 734 nm using a Shimadzu UV-1800 UV-Vis Spectrophotometer (Shimadzu Corporation, Kyoto, Japan). Antioxidant activity was calculated using a Trolox calibration curve (0–50 mg/g; y = 0.0001728x, R^2^ = 0.9832) and the results were reported as micrograms of Trolox equivalents per gram of dry weight (µg TE/g dw).

#### 4.4.3. DPPH Radical Scavenging Activity Assay

2 mL of DPPH solution (0.1 mM in ethanol) was combined with 2 mL of the sample, incubated in the dark for 30 min, and its absorbance was measured at 517 nm using a Shimadzu UV-1800. The results are expressed as micrograms of Trolox equivalents (µg TE/g dry weight), determined from a Trolox calibration curve (0–50 mg/g; y = 0.00623x; R^2^ = 0.9923).

### 4.5. Production of the Experimental Gel Formulations

Six control gels were formulated (Table 4). The selected gelling agents were xanthan gum and glucomannan. The gelling agent or their mixture was combined with glycerol, followed by the gradual addition of water. To the prepared mass, a 1% stevia solution (sweetener), a 10% vitamin C solution, and either ethanol or extract, along with a 1% potassium sorbate solution (preservative), were added.

### 4.6. Analysis of the Gels

#### 4.6.1. Gel’s pH and Stability

The pH value of the gels is determined potentiometrically using Orion VersaStar (“Thermo Scientific”, JAV) pH-meter. A 5% gel solution is prepared, and the pH is measured using a pH electrode. The analysis is repeated three times, and the arithmetic mean is calculated.

Preliminary stability testing was conducted for up to 2 months under refrigerated (6–8 °C) and room temperature (20 ± 5 °C) conditions, protected from light.

#### 4.6.2. Texture Analysis of Gels

Texture properties were evaluated using a TA.XT Plus Texture Analyzer (Stable Micro Systems, Godalming, UK). The measured parameters included firmness, consistency, cohesiveness, and viscosity index. The back extrusion technique, which is suitable for semi-solid formulations, was applied for all measurements.

The testing parameters were set as follows: pre-test speed 1.5 mm/s, test speed 2 mm/s, post-test speed 2 mm/s, penetration distance 10 mm, and trigger force 30 g. Gel samples were transferred into containers and analyzed under these specified conditions. Each formulation was analyzed in triplicate, and the mean values were used for further evaluation.

### 4.7. Cell Line and Cell Culture

Human bronchial epithelial cells (HBEC-3) were obtained from LGC Standards (Teddington, UK) and maintained in EpiLife medium supplemented with growth factors, 10% fetal bovine serum, 100 U/mL penicillin, and 100 µg/mL streptomycin. Cultures were incubated at 37 °C in a humidified atmosphere with 5% CO_2_. For experiments, cells were seeded into 24-well plates at a density of 20,000 cells per well, 24 h before the treatment.

### 4.8. Evaluation of Cell Viability

To evaluate the effect of the extract and formulation on cell viability under pathological conditions, HBEC-3 cells (passage numbers 6–8). To establish the in vitro damage model, HBEC-3 cells were exposed to different concentrations of LPS (100 nM, 500 nM, and 1 µM). Based on preliminary viability assessment, 1 µM LPS was selected for subsequent experiments, as it induced a statistically significant reduction in cell viability compared to lower concentrations. The HBEC-3 cells were treated with 1 µM LPS for 24 h in the presence of different concentrations of the test substances. After treatment, nuclear staining was performed by adding propidium iodide (PI, 3 µg/mL) and Hoechst 33342 (6 µg/mL) to the culture medium, followed by incubation for 5 min at 37 °C.

Cells were then visualised using a fluorescence microscope (Olympus IX71SIF-3, Olympus Corporation, Tokyo, Japan) at a magnification of ×320. Nuclei stained only with Hoechst 33342 (blue) were classified as viable; those double-stained with Hoechst 33342 and PI (magenta) as necrotic; and small condensed nuclei with intense blue fluorescence as apoptotic. Image analysis was performed using ImageJ software (version 1.53, National Institutes of Health, Bethesda, MD, USA; available at: https://imagej.nih.gov/ij/, accessed on 16 January 2023).

### 4.9. Statistical Analysis

Results are presented as the means of 3 experiments (performed in three technical replicates) ± standard error. Statistical data analysis was performed using Microsoft Excel software. Descriptive statistics included the calculation of means and standard deviations. Statistical significance was assessed using appropriate statistical tests, and results were considered statistically significant at *p* < 0.05. Differences between groups were evaluated using one-way analysis of variance (ANOVA), followed by Tukey’s post hoc test for multiple comparisons. For comparisons between two groups, a two-tailed Student’s t-test was applied.

## Figures and Tables

**Table 1:** TPC and antioxidant activity of elderflower extract.

Sample	TPC, mg GAE/g	FRAP, mg Fe(II)/g	ABTS, mg TE/g	DPPH, mg TE/g
Extract	197.746 ± 10.538	52.81 ± 2.93	28.389 ± 0.835	1.181 ± 0.015 mg/g

**Table 2:** Compounds determined in the elderflower extract according to their concentration.

No.	Compound	µg/mL	No	Compound	µg/mL
1	Chlorogenic acid	3768.16	7	3,5-di-caffeoylquinic acid	451.46
2	Rutin	3127.24	8	Astragalin	434.70
3	Isoquercitrin	634.25	9	4-O-caffeoylquinic acid	374.63
4	3,4-di-caffeoylquinic acid	577.07	10	4,5-di-caffeoylquinic acid	192.29
5	Nicotiflorin	574.37	11	Quercetin	108.65
6	Neochlorogenic acid	490.12	12	Protocatechuic acid	47.40

**Table 3:** Gel with elderflower extract texture parameters.

Sample	Firmness (g)	Consistency (g·sec)	Cohesiveness (g)	Index of Viscosity (g·sec)	pH Value
CP6 + extract	78.34 ± 4.49	743.67 ± 14.21	14.04 ± 3.44	54.31 ± 22.82	4.46 ± 0.15

**Table 4:** Composition of gel.

Ingredients	Gel Base Composition, g			Gel Base Composition with Preservative, g			Gel with Extract, g
	C1	C2	C3	CP4	CP5	CP6	G
Xanthan gum	0.5	0	0.25	0.5	0	0.25	0.25
Glucomannan	0	0.5	0.25	0	0.5	0.25	0.25
Vit. C 10% solution	5.0	5.0	5.0	5.0	5.0	5.0	5.0
Extract	-	-	-	-	-	-	5.0
Stevia 1% solution	1.0	2.0	2.0	2.0	2.0	2.0	2.0
Glycerol	1.0	1.0	1.0	2.0	2.0	2.0	2.0
Purified water	87.5	86.5	86.5	78.0	78.0	78.0	78.0
Ethanol 50%	5.0	5.0	5.0	5.0	5.0	5.0	-
Potassium sorbate 1% solution	-	-	-	7.5	7.5	7.5	7.5

## Data Availability

Data are contained within the article.

## Abbreviations

The following abbreviations are used in this manuscript:

FRAP	Ferric Reducing Antioxidant Power
TPC	Total Phenolic Compound
LPS	Lipopolysaccharide
HPLC–PDA	High-Performance Liquid Chromatography with Photodiode Array Detection
HBEC-3	Human Bronchial Epithelial Cells
